# Editorial: Integration of HIV prevention with sexual and reproductive health services

**DOI:** 10.3389/frph.2023.1129881

**Published:** 2023-01-23

**Authors:** Thesla Palanee-Phillips

**Affiliations:** ^1^ School of Public Health, University of the Witwatersrand, Wits Reproductive Health and HIV Institute, Johannesburg, South Africa; ^2^ Department of Epidemiology, School of Public Health, University of Washington, Seattle, WA, United States

**Keywords:** HIV prevention, sexual and reproductive health services, integrated healthcare, PrEP, STIs

Editorial on the Research Topic Integration of HIV prevention with sexual and reproductive health services

Sub-Saharan Africa (SSA) has the highest burden HIV infection globally, and over 57% of those infected are adolescent girls and young women (AGYW). AGYW of reproductive age in this region are at heightened risk of HIV infection, unplanned pregnancy and sexually transmitted infections (STI) due to a host of socio-behavioural, contextual, gender and relationship dynamics compounded by challenges related to access to sexual reproductive health (SRH) services. Annually, more than 200 million women globally experience unplanned pregnancies due to lack of access, uptake or awareness of availability to reliable contraceptive methods. The vast majority, 70%–80% of these women, reside in SSA. Consequently, almost 50% of pregnancies are unintended and 35 million unsafe abortions occur annually in this region.

Despite efforts to scale up SRH initiatives for the general population, progress made to reduce rates of HIV amongst the most marginalised groups are sub-optimal and high infection rates persist. As highly effective HIV prevention methods including treatment as prevention, HIV pre-exposure prophylaxis (PrEP), condoms and male circumcision have been available for more than a decade now, there are increasing challenges related to measuring impact of a single HIV prevention effort as combination prevention is usually recommended. Furthermore, evidence of pockets of HIV micro-epidemics (1) exist, reflecting hotspots of increased infection rates. AGYW exist in a socioecological matrix where multiple levels of influence exist leading to complex interplay between an individual, their relationships, community, and environment (2) when it comes to health seeking ability. Low perceived risk of HIV acquisition, side effects of PrEP/STI management, disapproval sexual partners, intermittent sexual acts, stigma, intimate partner violence, and perceived limited access to services reflect critical barriers to uptake of SRH care. While some programs have focused on oral PrEP uptake and refills, others adopt a more holistic approach, including counselling on risk reduction, contraceptives and condom use. While PrEP initiation among AGYW is high in some settings, known barriers of intermittent and poor adherence paired with high discontinuation rates reflect lack of sustained efforts to reduce HIV risk.

Aside from HIV, STIs are underdiagnosed in public healthcare facilities due to clinical management being driven by a syndromic symptom-based approach in SSA. Often asymptomatic or having non-specific symptoms, STIs are often unmanaged, increasing HIV transmissibility. Data from ECHO conducted among women seeking family planning (FP) services in HIV burdened settings in Africa demonstrated high HIV incidence of 3.8 per 100 woman-years with higher incidence in some community settings (1, 3). In addition, genitourinary STI rates among the same cohort from baseline through to trial completion remained high with baseline chlamydia and gonorrhoea prevalence rates at 18% and 5%, respectively (4) and final visit rates at 15% and 5% - a reflection of reinfection and persistence of infection despite initial lab diagnosed management and treatment on entry to the trial.

The World Health Organization (WHO) recommends that SRH services, including contraceptive method delivery, be integrated within HIV prevention and care services. Integration is associated with increased offers and uptake of SRH services, including contraceptive uptake, and STI services and reducing unwanted pregnancies, perinatal HIV transmission and maternal and infant mortality among people living with or without HIV. It is envisaged an integrated SRH model would improve equitable access, yield holistic and comprehensive care, raise the quality of maternal and antenatal care, be cost-effective to the client and the health system, increase financial sustainability with co-location of services and diversify healthcare provider capacity. Most critically, it would reduce stigma being a barrier to access due to shifts away from siloed care. With scale-up of oral PrEP and multiple novel HIV/STI prevention products on the horizon, it would offer a unique opportunity to expand innovative approaches to deliver comprehensive, integrated HIV prevention/SRH services.

However, despite the WHO call and theoretical support for integration, SRH services continue to remain predominantly distinct in most African countries. This may be attributed to the impact of legacy siloed structures that are challenging to modify due to lack of widespread funding or national ministries of health support, need for investment in provider training to support shifts to integration and concerns related to sustainability in this new model. Despite these limitations, research efforts continue to be encouraged to identify approaches for streamlined integration of SRH services. In an ideal world, provision of a comprehensive SRH programme should incorporate five major components: maternal and newborn health; family planning; prevention of unsafe abortion; management of reproductive tract infections and STIs, including HIV/AIDS; and promotion of sexual health all integrated within one discrete cohesive health care facility.

Researchers continue to launch and refine programs to provide evidence of best practices to inform wider scale-up and implementation. These programs span the breadth of HIV care – from testing to prevention to treatment and ongoing management – and leverage the breadth of SRH services – for pregnancy, family planning, and STI prevention and management – to offer myriad opportunities for client-centred, efficient, and comprehensive care. HIV/SRH service integration must be built on evidence of best practice implementation by those who have made efforts for better efficiency in processes. In order to showcase such evidence, a special Research Topic focussing on “Integration of HIV Prevention with SRH Services” was opened and invited submissions for consideration. Two guest editors facilitated the solicitation, peer-review and publication of manuscripts from multiple studies. A total of 14 manuscripts were received; one was rejected and 13 accepted for publication post peer review informed updates were made. By January 2022, the series achieved over 14, 000 views. The breadth of coverage within the series showcased evidence from across Africa and the United States and assesses ongoing integration efforts from different perspectives.

Publications include desktop scoping and landscape analyses across a number of African countries offering HIV testing services and PrEP delivery with family planning (FP) and SRH services to AGYW within the public healthcare space, in programmes and research studies (Drake et al., Mugwanya et al., Pleaner et al., Kasaro et al.) to a focus on the structural and motivational challenges faced by healthcare providers delivering PrEP alongside SRH services (O' Malley et al.). There was also focus on design of PrEP-FP integration matrices and assessing country-specific progress to identify common enablers of and barriers to PrEP-FP integration but also propose the matrix use as a potential roadmap to guide work towards more efficient achievement of integration (Bhavaraju et al.). Utilizing access to AGYW FP services attendees, Nyaboe et al. sought to better understand young women's risk profiling using contraceptive option selection as a proxy to determine risk categorisation when assessed alongside behavioural risk factors and how they may infer potential preferences for a range of short and long term PrEP delivery modalities. There was also evidence of a step further in integration beyond services to a focus in on products with dual or multiple indications within one modality offering both HIV/STI and pregnancy prevention (Friedland et al. and Young Holt et al.). These were recognised conceptually as options that are appealing with potential to revolutionise women's health – all with balance to also recognising opportunities and challenges that would accompany multi-indication product delivery and roll-out. The potential impact and appetite for discrete products with multiple indications would offer an alternate but very appealing integration of SRH services for AGYW who may face challenges disclosing use of methods for indications often stigmatised (e.g., HIV/STI prevention).

Overall, the evidence suggests more is needed to support SRH integration. Despite integration being theoretically and conceptually sound, there is insufficient evidence from the real world to demonstrate long term impact and benefit due to low uptake. Purely offering services in a facility does not speak to actual process integration and natural seamlessness of routine care. A standardised offering with opt-out options vs. opt-in may be useful to inform better SRH care. Evidence indicates that a one-stop women centred care approach would provide holistic care and reduce burden on the service dispersed models. One caveat remains; lack of adequate self-assessed risk awareness. AGYW generally have inaccurate and often low perceptions of their own risk. Design of age appropriate risk assessment tools with repeated opportunities for use will ultimately impact individual uptake of SRH services decreasing incidence of unplanned pregnancies, HIV and STIs.
